# Protective effect of protein hydrolysates from *Litopenaeus vannamei* waste on oxidative status, glucose regulation, and autophagy genes in non-alcoholic fatty liver disease in Wistar rats

**DOI:** 10.22038/IJBMS.2022.62167.13761

**Published:** 2022-08

**Authors:** Mirhossein Hadavi, Ebrahim H. Najdegerami, Mehdi Nikoo, Vahid Nejati

**Affiliations:** 1 Department of Biology, Faculty of Science, Urmia University, Urmia, Iran; 2 Department of Pathobiology and Quality Control, Artemia & Aquaculture Research institute, Urmia University, Urmia, Iran

**Keywords:** Autophagy, Bioactive peptides, Fatty liver, Insulin, Oxidative enzymes, Whiteleg shrimp

## Abstract

**Objective(s):**

The effects of protein hydrolysates (FP) from *Litopenaeus vannamei* on oxidative stress, and autophagy gene expression was investigated in the NAFLD-induced rats.

**Materials and Methods:**

For this purpose, twenty-four male rats were divided into four groups: Control, High-fat diet (HFD), FP20+HFD, and FP300+HFD (20 and 300 mg FP /kg rat body weight) and fed for 70 days.

**Results:**

The results indicated that the rat body and relative weight of the liver were not affected by experimental treatments (*P*>0.05) although the highest relative weight of the liver was observed in HFD treatment. The highest and lowest values for antioxidant enzymes and MDA concentration were observed in FP treatments (*P*<0.05). Also, the results showed that FP significantly decreased liver enzymes (ALT, AST) in the liver in comparison with HFD treatment (*P*<0.05). Plasma biochemical indices were investigated and the lowest amylase, ALP, fasting glucose, insulin, HOMA-IR, triglycerides, cholesterol, and inflammation cytokines (TNF-α, IL-6) were seen in the FP treatments which had a significant difference with HFD (*P*<0.05). Autophagy gene expression in the liver cells was affected by experimental diets and the lowest expression of Beclin-1 and Atg7 was observed in HFD and FP300 treatments. Interestingly, the highest expression of LC3-ɪ and P62 was seen in HFD and FP treatments, not in the control.

**Conclusion:**

Overall, the results of this experiment indicated that FPs extracted from Whiteleg shrimp at 50 °C improve the oxidative status, glucose metabolism, and autophagy gene expression and could be used as a useful nutritional strategy in fatty liver prevention.

## Introduction

Non-alcoholic fatty liver disease (NAFLD) is the most common liver disease affecting 25% of the world’s population. It is characterized by an increase in triglycerides and intrahepatic free fatty acids content without alcohol consumption (1). This disease, which is very common in developed countries due to improper diets, is associated with increased blood triglyceride levels after consumption of calories and lipids in the body (2). Therefore, NAFLD is often linked to obesity and dyslipidemia and can lead to liver fibrosis, cirrhosis, and liver cell cancer (3). Moreover, it is associated with complications such as insulin resistance, type 2 diabetes, hyperlipidemia, and high blood pressure (4). Other factors contributing to this disease include abdominal surgery such as gallbladder removal, surgery on the pancreas, removal of a part of the intestine or stomach, and the use of some medications, like estrogen and corticosteroids such as steroids, amiodarone, tamoxifen, and sodium valproate. So far, few treatments such as insulin sensitizers, lipid-lowering drugs, and Pentoxifylline have been recommended to treat NAFLD. However, these drugs have undesirable side effects including weight gain, nausea, vomiting, and higher mortality limiting their use (5). Therefore, scientists have been motivated to find methods based on using natural anti-oxidants.

Based on global statistics, the aquaculture industry generates huge amounts of protein-containing by-products that can be enzymatically broken down into smaller bioactive peptides. The latter substances can possibly be applied as additives to nutraceuticals and functional foods targeted for patients with NAFLD. Protein hydrolysates or bioactive peptides contain 2 to 20 amino acids with a molecular weight of 200 to 1800 DA and are normally composed of different amino acid sequences (6). The specific positions of these amino acids in the peptide chain determine the bioactive properties of the protein hydrolysates (6). As a result of bioactive properties, free forms of these peptides have several physiological functions such as immune system stimulation (7), antimicrobial effects (8), anti-oxidant properties (9), antihypertensive (3), and anti-inflammatory features (9). Several studies indicated that protein hydrolysates modulate complications in patients with NAFLD. A 2016 study revealed that the use of the VHVV peptide reduces the levels of low-density triacylglycerol, lipoprotein cholesterol, as well as liver cell fibrosis (10). Furthermore, in 2015 it was found that peptides extracted from salmon waste affect fat metabolism and reduce the synthesis of fatty acids and expression of lipogenic genes (11). Similarly, Huang *et al*. (2018) indicated that oligopeptides extracted from oysters (*Meretrix meretrix*) increased the expression of AMPk and PPAR genes and reduced the expression of SREBP-lc (12). This ultimately reduced the secretion of ALT and AST enzymes, and the concentration of MDA in rats with NAFLD. A study (2020) demonstrated that the use of protein hydrolysate extracted from Anchovy (*Engraulis encrasicolus*) waste reduced total cholesterol, serum triglycerides, liver enzyme activity, and liver triacylglycerol levels (13).

Whiteleg shrimp (*Litopenaeus vannami*), the most important commercial species of shrimp, is produced about 5 million tons per year (14). Since its production includes 50% waste from different parts of the body, using this capacity to extract bioactive substances can help the pharmaceutical industry (15). According to the results of the above studies, the present study aimed to investigate the effects of protein hydrolysates extracted from *L. Vannamei* waste at fixed temperature (50 °C) on liver anti-oxidant indices, and expression of autophagy-related genes in induced NAFLD male rats.

## Materials and Methods

***Peptide preparation***

**Figure 1:**
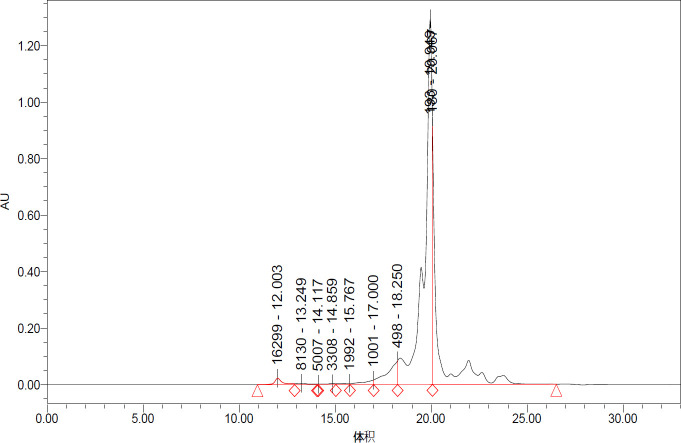
MWD of Pacific white shrimp hydrolysates obtained by alcalase hydrolysis. Enzyme: Substrate ratio was 5% for 180 min at 50 °C

Pacific white shrimp waste was purchased from a shrimp processing center (Persian Gulf Daryahodeh Co., Bushehr, Iran). By-products were minced with a meat grinder using a 3 mm hole plate (Pars Khazar Co., Tehran, Iran) and then mixed at a 1:1 ratio with distilled water and homogenized for 2 min with a Heidolph DIAX900 homogenizer (Heidolph Instruments GmbH, Schwabach, Germany). Hydrolysis was done by Alcalase enzyme and according to the method of Nikoo *et al*. (2021) at an initial pH (~7.1) using fixed temperature (FT) (50 ºC) for 3 hr (16). During autolysis, the mixture was continuously stirred using an overhead stirrer. The mixtures were heated in a boiling water bath (~95 °C) for 10 min to stop the reaction, filtered using two layers of cheesecloth followed by centrifugation at 4000 × g for 20 min at 4 °C and the supernatants were freeze-dried. The molecular weight distribution of produced protein hydrolysates was determined and the results indicated that the peptides with varying chain length were generated following autolysis and the percentage of < 500 Da was 40% of total peptides in the protein hydrolysate (16) (Figure 1).

***Animals, diets, and experiment design***

Twenty-four male Wistar rats with an initial average weight of 230.2±23 g were prepared from the animal house of the Department of Biology, Urmia University, and divided into four cages (6 rats per treatment). The rats were fed a standard diet during the adaptation period at 25 °C, and a 12 hr-light regime. After one week of adaptation, the animals were divided into four experimental treatments (Control: standard diet, HFD: High-fat diet, FP20: High-fat diet+20 mg/kg peptide per body weight of rat, FP300: High-fat diet +300 mg/kg of peptide per body weight of rat) (5, 17). To prepare a high-fat diet, 10% animal fat and 5% fructose were added to 85% of normal diets (18). Bioactive peptide solution at concentrations of 20 and 300 mg/kg body weight of the rats were freshly prepared and dissolved in 4 ml of distilled water daily and entered the gastrointestinal tract of the rats by an oro-gastric feeding needle. It should be noted that a standard diet was used for the control group and 4 ml distilled water was administered orally using an oro-gastric feeding needle.

***Blood collection and liver sampling***

After 70 days, the rats were anesthetized to avoid any stress during blood sampling. After weighing, the blood samples were taken directly from the animal’s heart with 5-mm syringes impregnated with heparin anticoagulant. The plasma of blood samples was isolated after centrifugation at 3500 g for 10 min. The samples were kept at -80 °C to measure inflammatory cytokines (TNF-α and IL6) and other plasma indices. The autopsy was performed after blood sampling, and the liver sample of the rats was divided into two parts after weighing. The samples were taken to -80 °C for measuring the anti-oxidant enzymes, and expression of autophagy genes. The second part was fixed in 10% formalin for histological examinations.

***Biochemical evaluations***

*Measurement of anti-oxidant enzymes in the liver*

One gram of the liver tissue kept at -80 °C, was homogenized at a temperature close to 0 °C at a 1:10 ratio with physiological serum for 1 min using a homogenizer (homogenization for 20 sec, stop for 5 sec). Finally, the mixture was transferred to the micro-centrifuge and centrifuged (20000 rpm, 4 °C) for 10 min. The supernatant was separated and transferred to a new microtube to evaluate the anti-oxidant indices (TAC, GSH, SOD, and MDA concentration) by using Arsam Farazist kits (Arsam Farazist, Urmia, Iran). Reduced Glutathione (GSH) was assessed using the thiol concentration (yellow) in glutathione which reacts with the element reagent namely dinitrothiocyanatebenzene (DNTB) and produces nitro thiobenzoate (TNB). The produced yellow color was quantified at 412 nm. The color intensity was directly proportional to the reductive thiol. Finally, the amount of GSH in mmol/mg of protein was expressed (19). Lipid peroxidation is one of the cell damage mechanisms in animals and plants that can be measured by malondialdehyde (MDA). The TBARS test is a direct quantitative method to measure MDA. The samples and MDA standards first react with TBA at 95 °C and MDA concentration was expressed based on nmol of MDA per mg of protein (20). Total anti-oxidant capacity (TAC) was measured using the ABTS method which can be oxidized to ABTS^+^ (green) in the presence of a suitable oxidant, and anti-oxidants inhibit the process. The TAC of the samples can be determined by measuring ABTS^+^ absorption at 414 nm. To measure SOD, the method developed by Sun *et al*. (1988) was used (21). The amount of enzyme activity is directly related to the degree of inhibition of oxidation of Nitroblue tetrazolium by O_2_^-^ anion. The absorbance was read at 580 nm, and the enzyme activity was expressed as a unit in mg of protein. It is worth noting that the amount of supernatant protein was measured according to the method of Lowry (1951) and bovine serum albumin was used as the standard (22).

*Measuring the biochemical indices and pro-inflammatory factors in plasma*

Commercial kits of Darman Faraz Kave (Isfahan, Iran) were used to measure amylase, alkaline phosphatase, liver enzymes (ALT and AST), cholesterol, and triglyceride concentration. To measure amylase activity, the amount of produced p-nitrophenol was read at 405 nm, which is directly related to amylase activity and reported according to the kit protocol. The alkaline phosphatase separates the phosphate group from 4-nitrophenol phosphate and produces 4-nitrophenol, which is colorless in a weakly acidic environment. Under alkaline conditions, 4-nitrophenol forms a yellow phenoxide ion readable at 405 nm. 

AST and ALT were measured according to the commercial kit protocol and their concentration was reported in Unit/l. To measure triglycerides, we used the mechanism of its conversion to hydrogen peroxide by lipase, glycerokinase, and glycerol phosphate oxidase which produces colorful Quinoneimine by peroxidase in the presence of 4-aminophenazone and 4-chlorophenol. The Quinoneimine concentration was read at 500 nm and the amount of triglyceride was reported in mg/dl. To calculate the cholesterol concentration, the formation of Qunioneimine using hydrogen peroxide in the presence of 4-aminophenazen and 4-chlorophenol was used. The amount of this color was read at 520 nm which is directly proportional with the amount of plasma cholesterol (mg/dl). Also, at the end of the experiment, the levels of pro-inflammatory factors such as TNF-ɑ and IL-6 in plasma were calculated, using commercial kits (Zelbio, Germany) according to the kit protocol.

*Determination of fasting blood glucose, glucose tolerance, insulin, and HOMA-IR*

To measure fasting glucose concentration and glucose resistance, the animals were deprived of food for 14 hr but had free access to water (23). The blood samples were taken through the tail vein and the glucose concentration was measured and reported using a commercial kit (Darman Faraz kave, Esfahan, Iran). Resistance of the rats to glucose was determined by glucose solution (2 g/kg body weight of the rats), and its administration was orally using an oro-gastric feeding needle. The blood glucose level was measured using a glucometer at 0, 30, 60, 90, and 120 min, for which a graph of glucose changes was plotted for all treatments. For calculation of HOMA-IR, the method of Matthews *et al*., (1985) (Insulin (µIU/ml) × fasting glucose (µmol/L)/22.5) was used (24). We measured plasma insulin levels using a kit from Zelbio Company (Berlin, Germany) and the ELISA technique.

*Analysis of mRNA expression of autophagy genes by RT-qPCR*

The TRIZOL method was used to extract mRNA to investigate the expression of autophagy genes, including Beclin 1, Atg7, LC3- ɪ, and P62 in the rat liver. To this end, 20–30 mg of liver tissue was homogenized using TRIZOL solution. After total extraction of mRNA, its quantity and quality were measured by NanoDrop spectrophotometer (260 nm). RNA with quality of more than 1.8–2 was considered for the synthesis of cDNA. Then, cDNA was synthesized in the reaction mixture of 20 ml containing 1 mg RNA, OLIGO Primer (1 μl), reaction buffer (4 μl), RNAse inhibitor (1 μl), 10 mM of dNTP mixture (2 μl), and M-MuLV reverse transcriptase (1 μl) according to the manufacturer’s protocol (Pars Tous Company, CAT: A101161, Iran). We also performed quantitative RT-qPCR tests for each sample using a thermal mini-cycler MyGo PCR (USA) in three versions. qPCR reaction mixtures contained 0.5 μl of the cDNA pattern, 10 μl of 2˟SYBER GREEN master (High ROX, Noavaran Teb Beynolmelal company, Iran), and 0.5 μl of forward and reverse primers of the target genes. Specific primers were selected using Multiple alignment program for amino acid or nucleotide sequences (MAFFT) V.7 (https://maﬀt.cbrc.jp/alignment/server) which was designed and built by Gen Fanavaran Company (Tehran, Iran). Table 1 presents the primer pair sequence for each gene. The thermal cycling conditions of qPCR are as follows: a general denaturation at 95 °C for 5 min followed by 40 denaturation cycles at 95 °C for 20 sec, annealing (Table 1) for 30 sec , and continuing at 72 °C for 30 sec . The average values of Ct (threshold cycle) were normalized from triple readings of each gene with an average CT value of the internal control gene (GAPDH) and the relative expression level of each gene was calculated using the ΔCt method: 2^− ^(dCt gene of interest − dCt internal control gene) (25).

*Preparation and analysis of liver tissue*

For liver histological investigations, fixed samples in 10% formalin were used. Liver sections (5 μm) were stained with hematoxylin & eosin and Sudan Black B using standard techniques, and fat accumulation in liver sections was observed by the method of Brunt *et al*. (1999) (26).

***Statistical calculations***

Data are presented as mean ± standard deviation (SD) for n = 6 rats per treatment. The data were examined for normality (Kolmogorov – Smirnov test) and homogeneity of variance (Levene’s test), then one-way ANOVA and Kruskal-Wallis tests were used to compare the means. Excel software V. 2013 was used to draw graphs and Spss software V.21 was applied to examine statistical changes in research treatments.

## Results

***Weight gain and relative weights of livers***

The results of the effects of protein hydrolysate on weight gain and relative weights of livers are presented in Table 1. Based on these results, the rats fed experimental diets did not reveal differences between experimental treatments in the case of body weight and relative weights of livers (*P*<0.05), although the highest relative weight of the liver was observed in HFD treatment. 

***Oxidative status and serological parameters***

**Figure 2:**
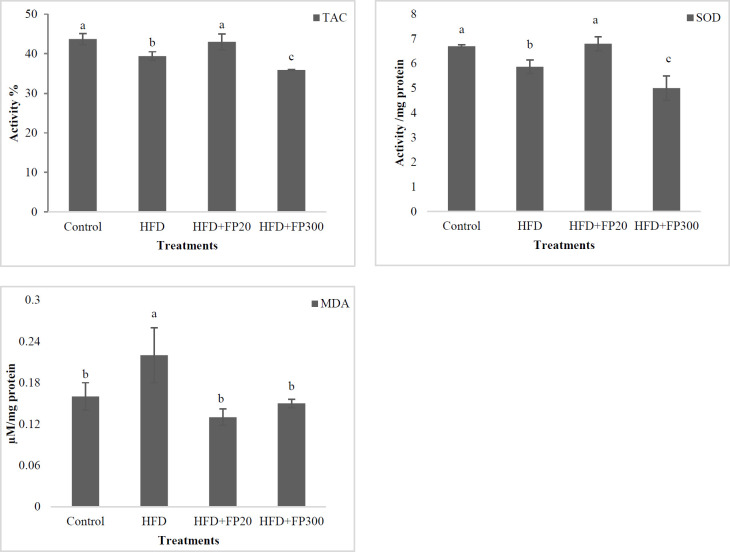
Effects of protein hydrolysates on TAC, SOD, and MDA concentrations in liver tissue in induced NAFLD rats. Data are presented as the mean ± SD (n = 6 per treatment). Results were statistically analyzed using one-way ANOVA followed by Duncan's multiple-comparison test, and values with different labels (a–c) are significantly different (*P*<0.05)

At the end of the experiment, liver oxidative status was evaluated in NAFLD-induced rats and the results are presented in Figure 2. The results showed that the use of the protein hydrolysates affected the TAC and the highest values were observed in the control and HFD+FP20 treatments which differed from the others (*P*<0.05). Also, the rats fed the HFD and high dose of the peptides showed a low level of TAC, and the lowest value was seen in HFD+FP300 which had a significant difference with HFD (*P*<0.05). A similar pattern was observed for SOD enzyme activity as seen for TAC. The concentration of MDA was increased significantly when the rats were fed HFD treatment as compared with the others (*P*<0.05). No significant difference was seen between control and peptides treatments (*P*>0.05).

**Figure 3:**
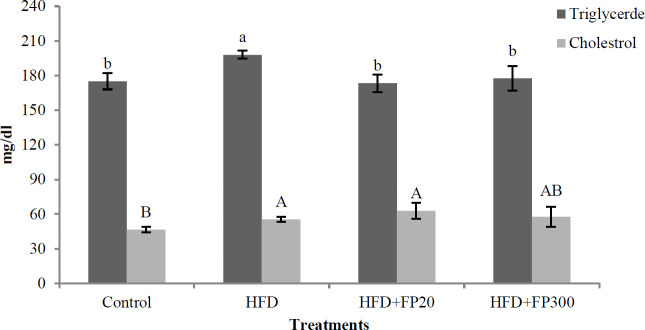
Effects of protein hydrolysates extracted from Whiteleg shrimp waste on serum triglyceride and cholesterol in induced NAFLD rats. Data are presented as the mean ± SD (n=6 per treatment). Results were statistically analyzed using one-way ANOVA followed by Duncan's multiple-comparison test, and values with different labels (a–c) are significantly different (*P*<0.05)

NAFLD: Non-alcoholic fatty liver disease; Control: Standard diet; HFD: High-fat diet; FP20: High-fat diet+20 mg/kg peptide per body weight of rat; FP300: High-fat diet +300 mg/kg of peptide per body weight of rat

Data in Figure 3, show the effect of the protein hydrolysates extracted from Whiteleg shrimp waste on serum triglycerides and cholesterol. The data showed that the rats fed HFD had a significantly higher triglyceride concentration when compared with the others (*P*<0.05). At the end of the experiment, no significant difference was seen between peptide treatments and control (*P*>0.05). Protein hydrolysates affected the cholesterol concentration and the rats fed the HFD and FP treatments (HFD + FP20, HFD +FP300) showed a significantly higher concentration in comparison with the control (*P*<0.05).

**Figure 4:**
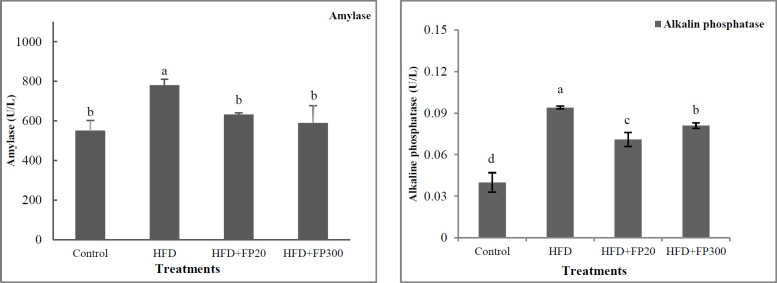
Effects of protein hydrolysates extracted from Whiteleg shrimp waste on serum amylase and alkaline phosphatase in induced NAFLD rats. Data are presented as the mean ± SD (n = 6 per treatment). Results were statistically analyzed using one-way ANOVA followed by Duncan's multiple-comparison test, and values with different labels (a–c) are significantly different (*P*<0.05)

NAFLD: Non-alcoholic fatty liver disease; Control: Standard diet; HFD: High-fat diet; FP20: High-fat diet+20 mg/kg peptide per body weight of rat; FP300: High-fat diet +300 mg/kg of peptide per body weight of rat

The serum content of the amylase and alkaline phosphatase activity are presented in Figure 4. HFD treatments were characterized by their higher amylase content as compared with those in the other treatments (*P*<0.05). No significant difference was found between control and FP treatments (*P*>0.05). All the rats fed HFD and FP treatments showed a significantly high alkaline phosphatase activity when compared with control (*P*<0.05). Furthermore, peptide treatments showed a significantly lower alkaline phosphatase activity as compared with HFD (*P*<0.05), and the lowest activity was observed in HFD+FP20 which differed from HFD300 (*P*<0.05). 

***ALT, AST, and pro-inflammatory cytokines***

**Figure 5:**
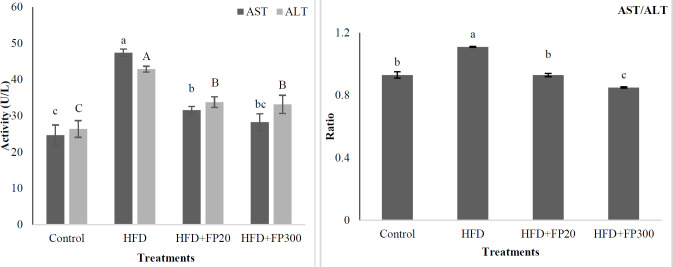
Effects of protein hydrolysates extracted from Whiteleg shrimp waste on serum ALT, AST and AST/ALT ratio in induced NAFLD rats. Data are presented as the mean ± SD (n = 6 per treatment). Results were statistically analyzed using one-way ANOVA followed by Duncan's multiple-comparison test, and values with different labels (a–c) are significantly different (*P*<0.05)

ALT: Alanine transferase; AST: Aspartate transaminase; NAFLD: Non-alcoholic fatty liver disease; Control: Standard diet, HFD: High-fat diet, FP20: High-fat diet+20 mg/kg peptide per body weight of rat, FP300: High-fat diet +300 mg/kg of peptide per body weight of rat

Figure 5 illustrates the data of serum ALT, AST, and AST/ALT ratio as affected by protein hydrolysates. The activity of ALT and AST increased significantly when the rats fed HFD treatment as compared with the other treatments (*P*<0.05). Also, the results indicated that feeding with shrimp protein hydrolysates significantly lowered serum ALT and AST levels as compared with HFD (*P*<0.05). However, the activity of both enzymes was lowest in the control which had a significant difference from the others (*P*<0.05). The AST/ALT ratio in the present study was calculated and the result is presented in Figure 5. Based on these results, the rats fed HFD had a significantly higher ratio than the other treatments (*P*<0.05). No significant difference was found between control and HFD+FP20 treatments (*P*>0.05), but feeding with a high dose of peptides decreased significantly this ratio (*P*<0.05).

**Figure 6:**
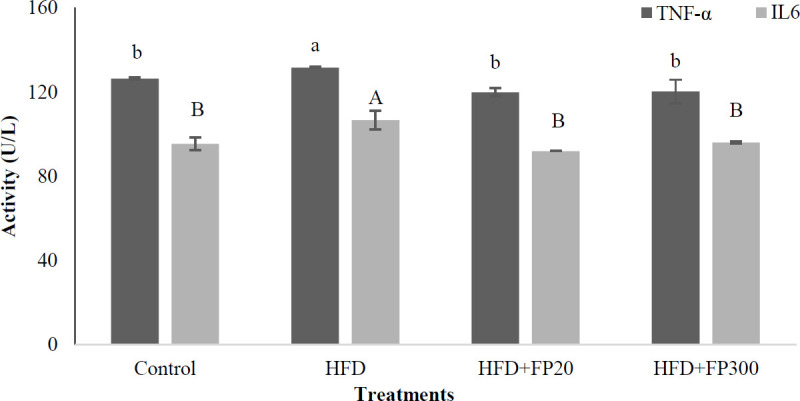
Effects of protein hydrolysates extracted from Whiteleg shrimp waste on serum pro-inflammatory cytokines (IL6 and TNF-ɑ) in induced NAFLD rats. Data are presented as the mean ± SD (n = 6 per treatment). Results were statistically analyzed using one-way ANOVA followed by Duncan's multiple-comparison test, and values with different labels (a–c) are significantly different (*P*<0.05)

The results of the effects of protein hydrolysates on serum pro-inflammatory cytokines are presented in Figure 6. Our findings indicated that the rats fed HFD showed significantly higher IL6 and TNF-ɑ levels in their serum than the others (*P*<0.05). Based on these results no significant difference was seen between control and FP treatments (*P*>0.05).

***Effects on fasting glucose, glucose tolerance, insulin secretion, and HOMA-IR***

**Figure 7:**
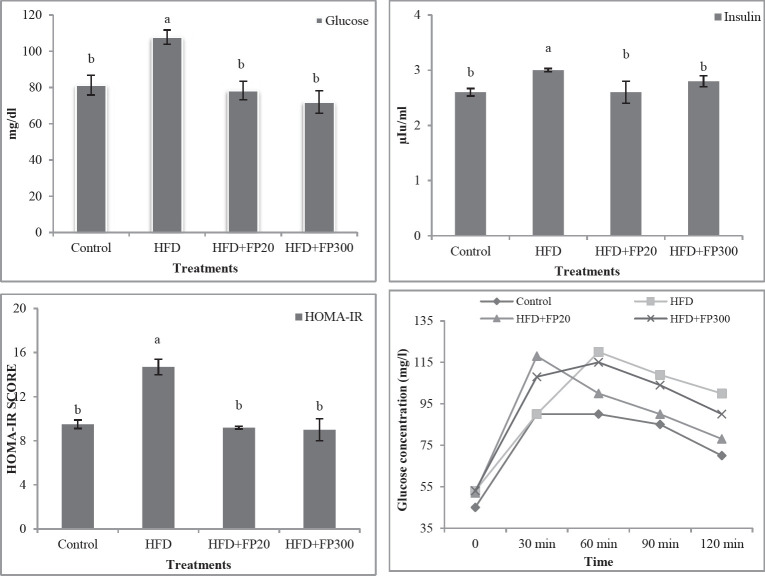
Effects of protein hydrolysates extracted from Whiteleg shrimp waste on serum fasting glucose, glucose tolerance, insulin secretion, and HOMA-IR in induced NAFLD rats. Data are presented as the mean ± SD (n = 6 per treatment). Results were statistically analyzed using one-way ANOVA followed by Duncan's multiple-comparison test, and values with different labels (a–c) are significantly different (*P*<0.05)

 Results, shown in Figure 7, revealed that HFD increased significantly glucose concentration, insulin secretion, and HOMA-IR in the rats’ serum when compared with the others (*P*<0.05). No significant difference was observed between the rats fed control and FP treatments (*P*<0.05). Also, the results indicated that HFD induced a hyperglycemic state in the experimental rats. Based on these results, HFD increased glucose tolerance while protein hydrolysates extracted from Whiteleg shrimp decreased the aforementioned parameter in the rats. The lowest glucose tolerance was observed in the control treatment. 

***Effects of HFD on the expression of liver autophagy genes; Becline 1, Atg7, LC*** ***3-ɪ,*** ***and P62***

To find the effects of HFD on liver autophagy, the alterations of Becline 1, Atg7, LC3-ɪ, and P62 expression were investigated in the rat’s liver. At the end of the experiment, the results indicated that the rats fed HFD and HFD+FP300 showed significantly lower Becline 1 expression when compared with control and HFD+FP20 treatments (*P*<0.05). The highest expression for Becline 1 was seen in HFD+FP20 which differed from control (*P*<0.05). Based on these results, feeding on HFD, HFD+FP20, and HFD+FP300 treatments significantly decreased Atg7 expression compared with the control (*P*<0.05). The lowest expression of Atg7 was seen in HFD+FP300, then HFD, and finally HFD+FP20, which were significantly different (*P*<0.05). Dietary treatments significantly affected the expression of LC3-ɪ and the highest values were observed in HFD and FP treatments which differed from control (*P*<0.05). No significant difference was seen between HFD and FP treatments (*P*>0.05). The rats fed HFD showed significantly highest expression of P62 among experimental treatments (*P*<0.05). Peptides significantly decreased the expression of P62, however, the lowest expression was found in the control.

## Discussion

Non-alcoholic fatty liver disease (NAFLD) is a common disease in different societies due to high-fat accumulation (5%) and inadequate triglyceride metabolism in liver cells (27). Excessive accumulation of lipids and oxidation of fatty acids (ω-oxidation) in various organelles such as mitochondria, cytochrome, and peroxisome causes the production of free radicals (ROS), oxidative stress, and toxic substances including dicarboxylic acids, eventually causing inflammation and disease progression (28-30). It also causes mitochondrial dysfunction and apoptosis which can damage liver cells and stimulate the immune system against free radicals (31).

**Figure 8:**
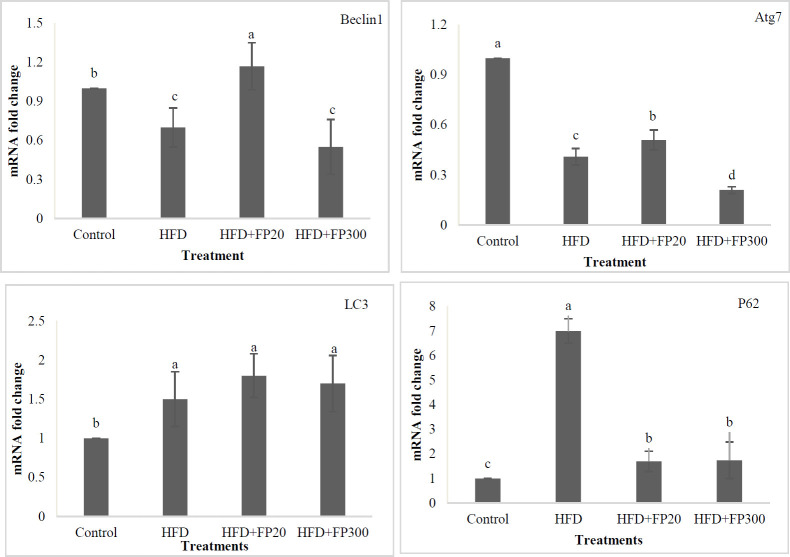
Expression of autophagy genes (Becline 1, Atg7, LC3-ɪ, and P62) in the liver of the induced NAFLD rats. Data are presented as the mean ± SD (n = 6 per treatment). Results were statistically analyzed using one-way ANOVA followed by Duncan's multiple-comparison test, and values with different labels (a–c) are significantly different (*P*<0.05)

In the present study, lipid accumulation in liver cells was confirmed through tissue results (Figure 8) in the HFD treatment. Moreover, the levels of triglycerides and cholesterol in this treatment were significantly higher than in control and FP treatments. This indicates the high accumulation of lipids in the most important regulatory organ of lipid metabolism in the body (32). According to Lemus-Conejo *et al*. (2020), bioactive peptides regulate enzymes related to triglyceride synthesis (33). They also increase lipoprotein lipase activity and fatty acid oxidation and reduce triglyceride concentration by suppressing the expression of fatty acid synthetic genes (34). It has been reported that there is a direct strong relationship between triglyceride concentration in plasma, liver enzymes (ALT and AST), and NAFLD (35). Therefore based on previous findings, an increase in liver enzymes was expected in the rats fed the HFD due to free radicals, cell damage, and lipid accumulation, which was another sign of liver cell damage in HFD treatment (36). 

The rats fed HFD treatments had lower anti-oxidant capacity, GSH, and SOD activity whereas the highest MDA concentration was observed in HFD. Dysfunction of mitochondria as metabolic organ plays a key role in the production of free radicals in NFLD eventually leading to inflammation, apoptosis, and increased oxidative stress (37). In this regard, GSH plays a crucial role in free radical elimination, and protein hydrolysates provide cysteine as a glutathione precursor thus reducing free radicals (38). Also, SOD as a leading enzyme plays an important role in eliminating free radicals and its reduced secretion in the HFD can be caused by a sharp increase in free radicals and its resultant continuous use in the cells. MDA is a product of lipid peroxidation in cells, and when increasing, it causes an inflammatory response, consequently, cell damage (39). Several studies reported that bioactive peptides reduce stress levels in different cells using various mechanisms including free radical scavenging, chelating, production of a stable product with electron donor, and finally by increasing the expression of some genes involved in oxidative stress (40). Ultimately, these functions reduce oxidative stress and MDA concentration.

Unlike FP treatments, the rats fed HFD had the highest concentration of alkaline phosphatase in their plasma. Various studies reported that different factors such as heat stress, hypoxia, and cell damage increase alkaline phosphatase (41). It should also be noted that alkaline phosphatase is a membrane hydrolysis enzyme and one of the main biomarkers of cholestasis due to hepatic steatosis and imbalance in the metabolism of lipids and carbohydrates. Eventually, when increasing, it leads to an increase in inflammatory factors such as TNF-ɑ and IL-6, resulting in insulin resistance in the body (42, 43). Burski *et al*. (2014) reported that amylase activity is known as an indicator of pancreas dysfunctions (44). In this study, the rats fed HFD showed a significant increase in amylase activity. The pancreatic function was not evaluated in the current study, and the relationship between increased amylase and pancreatic function needs further investigation. In contrast, FP treatments showed an inhibitory impact on amylase activity and it seems that protein hydrolysate may delay the carbohydrate metabolism and this may in part account for the weight loss observed in the rats fed HFD, affecting through the inhibition of carbohydrate metabolism (45). Also, it seems that bioactive peptides decrease the amylase activity when compared with HFD, it is possible that feeding on bioactive peptides improves pancreatic function (46).

In addition to oxidative stress, inflammation mainly contributes to liver disease and pro-inflammatory factors such as IL6 and TNF-α significantly increase fatty liver disease (5). The results of this study showed that the use of HFD increased TNF-α and IL-6 while bioactive peptides significantly reduced the aforementioned cytokines. Lemus-Conejo *et al*. (2020) indicated that pro-inflammatory cytokines decrease lipid degradation and lipolysis, resulting in lipid accumulation, which are the main reasons for the higher accumulation of lipids in the liver and plasma in HFD (34). Also, based on previous findings bioactive peptides (especially with low molecular weight) decrease free radicals produced from lipid metabolism and mitochondrial disorders by scavenging properties which help to improve the oxidative status and inflammatory responses (47). These results were consistent with our findings obtained in the section on oxidative stress and tissue observations in HFD and FP diets.

The results related to the effects of marine protein hydrolysates on glucose metabolism in NAFLD rodents are contradictory. Drotningsvik *et al*. (2016, 2015) reported that the feeding of obese rats with cod, salmon, and herring protein decreased 2-h postprandial glucose whereas it did not affect fasting glucose and insulin concentration, indicating that the aforementioned sources did not affect fasting glucose regulation (48, 49). In contrast, Sarteshnizi *et al*. (2021) revealed that protein hydrolysates extracted from sardines delay the hydrolysis of carbohydrates by inhibiting α-glucosidase and α-amylase, thus increasing its digestion time, and decreasing glucose uptake and insulin secretion (50). Also, a study (2015) reported that lipid accumulation in the liver stimulates the secretion of pro-inflammatory factors (TNF-ɑ, IL-6), thus reducing insulin signaling, which ultimately causes more lipid accumulation and insulin resistance (23). In the current study, the results of the fasting glucose and insulin concentration indicated that the rats fed protein hydrolysates had significantly lower fasting glucose and insulin secretion when compared with HFD treatment. Based on these results, glucose reduction was greater than that of insulin, indicating that glucose was reduced in ways other than insulin secretion. Also, Das *et al*. (2020) and Kulakowski *et al*. (1984) reported that the Taurine has hypoglycemic effect in rats and decreases glucose uptake without increasing insulin secretion (51, 52). To the best of our knowledge, taurine is an amino acid that is abundant in fish meals but limited in plant protein sources (53). Also, our results showed that the average decrease in glucose was significantly greater after 60 min in the 20 mg/BWkg treatments in comparison with 300 mg/BW kg, indicating an effective postprandial glucose regulation in low-dose protein hydrolysates.

Autophagy is an important intracellular mechanism in the homeostasis of cells and their long-term survival (25). This mechanism controls the amount and quality of cytoplasmic contents in eukaryotic cells, which includes digestion of damaged proteins and organelles, lipids, and carbohydrates (54). A set of factors such as oxidative stress, chronic inflammation, and lipotoxicity as well as response to autophagy suppression often increase hepatocyte cell death (55). In selecting medicines, efforts should be made to increase liver autophagy to reduce the progression of liver diseases involving inflammation or injury, including non-alcoholic fatty liver (NAFL) or non-alcoholic steatohepatitis (NASH) (56). Among the genes involved in autophagy, Beclin-1, Atg7, LC3-ɪ, and P62 genes play a major role in this process. Beclin-1 builds the primary autophagosomal nucleus in this process, Atg7 and LC3-ɪ complete the autophagosome wall with the help of some proteins (57). Gene P62 is the main and influential gene in autophagy and transfers the damaged organelles and lipid droplets to autophagosomes for decomposition and completes the autophagy process (58). In this study, the expression of the aforementioned genes was investigated, for which the results are presented in Figure 8. Based on the results, the lowest expression of Beclin-1 and Atg7 genes was observed in HFD treatment, indicating oxidative conditions, lipid accumulation, and lack of homeostasis inside the liver cells. The findings were confirmed by the results of anti-oxidant status, liver enzymes (ALT and AST), histopathological observations, and the presence of inflammatory factors in the treatment. Furthermore, the use of bioactive peptides, especially at low concentrations significantly increased the expression rates of Beclin-1 and Atg7 genes. Interestingly, the highest expression of LC3-ɪ and P62 was seen in HFD, FP20, and FP300 treatments not in the control. Koga *et al*. (2010) and Gonzalez-Rodriguez *et al*. (2014) reported that lipid changes in autophagosome membrane decrease vesicles’ ability to connect with lysosomes, resulting in reduction in the autophagy process. This reduction in autophagosome clearance could explain the accumulation of LC3-II and p62 observed in NAFL and NASH patients and is positively related to disease severity (59, 60). 

MWD: Molecular weight distribution

**Table 1:** Weight gain and relative weights of livers in induced Non-alcoholic fatty liver disease (NAFLD) rats after 70 days of the experiment period

	Control	HFD	HFD+FP20	HFD+FP300
Weight gain (g)	62.6 ± 11.2 ^a^	53.6 ± 10.4 ^a^	56.5 ± 7.3 ^a^	59.0 ± 5.5 ^a^
Relative weights of livers (g)	2.8 ± 0.2 ^a^	3.2 ± 0.2 ^a^	2.7 ± 0.3 ^a^	2.7 ± 0.2 ^a^

Data are expressed as mean ± SD (n= 6 per treatment)

Different letters within a row indicate significant differences (*P*<0.05)

**Table 2:** Nucleotides sequences of primers used for PCR

**Gene**	Primer	AT	Bp
P62	F: GCTGCTCTCTTCAGGCTTACAG	53°c	22
	R: CCTGCTTCACAGTAGACGAAAG		
Beclin-1	F: AGCACGCCATGTATAGCAAAGA	51°c	22
	R: GGAAGAGGGAAAGGACAGCAT		
Atg7	F: AGCCTGTTCATCCAAAGTTCT	46°c	21
	R: CTGTGGTTGCTCAGACGGT		
LC3-I	F: GATGTCCGACTTATTCGAGAGC	46°c	22
	R: TTGAGCTGTAAGCGCCTTCTA		
GAPDH	F: AAGGTCATCCATGACAACTT	58°c	20
	R: GGCCATCCACAGTCTTCTGG		

TAC: Total anti-oxidant capacity; MAD: Malondialdehyde; NAFLD: Non-alcoholic fatty liver disease; Control: Standard diet; HFD: High-fat diet; FP20: High-fat diet+20 mg/kg peptide per body weight of rat; FP300: High-fat diet +300 mg/kg of peptide per body weight of rat

Control: Standard diet; HFD: High-fat diet; FP20: High-fat diet+20 mg/kg peptide per body weight of rat; FP300: High-fat diet +300 mg/kg of peptide per body weight of rat

Control: Standard diet, HFD: High-fat diet, FP20: High-fat diet+20 mg/kg peptide per body weight of rat, FP300: High-fat diet +300 mg/kg of peptide per body weight of rat

Control: Standard diet, HFD: High-fat diet, FP20: High-fat diet+20 mg/kg peptide per body weight of rat, FP300: High-fat diet +300 mg/kg of peptide per body weight of rat

**Figure 9:**
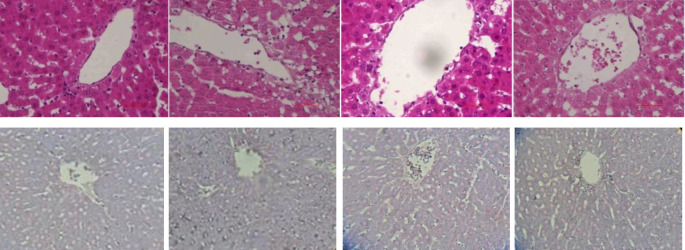
Hematoxylin and eosin (H&E) and (above) and Sudan black (down) staining of liver tissue sections from Control, HFD, HFD+FP20, and HFD+FP300 (left to right, respectively) (˟400)

HFD: High-fat diet

## Conclusion

The results of the current study showed that the protein hydrolysates extracted from *L. vannamei* waste did not affect the rats’ weight gain and liver weight. In contrast, protein hydrolysates improved oxidative status, liver enzymes, and pro-inflammatory factors in the rats fed the HFD. Also, the results indicated that protein hydrolysates regulate fasting glucose, postprandial glucose, and insulin concentration in NAFLD-induced rats. In addition, protein hydrolysates at low concentrations stimulate autophagy gene expression and help the liver cells to make homeostasis of the cells and their long-term survival. Based on these results and nutritional proprieties, protein hydrolysates extracted from *L. vannamei* waste could be used as a useful nutritional strategy in NAFLD treatment and prevention. 

## Authors’ Contributions

MH Contributed to the study conception, conducting the work, and drafting the manuscript. EH Contributed to the conception of the work, conducting the study, and revising and approval of the final manuscript (experiment design, data analysis, and gene expression). MN Contributed to conducting part of the experiments (peptides extraction and analyzing). VN Contributed to conducting part of the study (histological investigation).

## Ethical Approval

All animal experimental protocols were approved by the Animal Ethics Committee of Urmia University. 

## Conflicts of Interest

There is no conflicts of interest in this paper.

## References

[1] Fabbrini E, Sullivan S, Klein S (2010). Obesity and nonalcoholic fatty liver disease: Biochemical, metabolic, and clinical implications. Hepatology.

[2] Carabelli J, Burgueño AL, Rosselli MS, Gianotti TF, Lago NR, Pirola CJ (2011). High fat diet‐induced liver steatosis promotes an increase in liver mitochondrial biogenesis in response to hypoxia. J Cell Mol Med.

[3] Erdmann K, Grosser N, Schipporeit K, Schröder H (2006). The ACE inhibitory dipeptide Met-Tyr diminishes free radical formation in human endothelial cells via induction of heme oxygenase-1 and ferritin. J Nutr.

[4] Karlas T, Wiegand J, Berg T (2013). Gastrointestinal complications of obesity: non-alcoholic fatty liver disease (NAFLD) and its sequelae. Best Pract Res Clin Endocrinol Metab.

[5] Gao J, Song J, Du M, Mao X (2019). Bovine α-lactalbumin hydrolysates (α-LAH) attenuate high-fat diet induced nonalcoholic fatty liver disease by modulating hepatic lipid metabolism in C57BL/6J mice. JFF.

[6] Giri A, Ohshima T (2012). Bioactive marine peptides: nutraceutical value and novel approaches. Adv Food Nutr Res.

[7] Agyei D, Danquah MK (2012). Rethinking food-derived bioactive peptides for antimicrobial and immunomodulatory activities. Trends Food Sci Technol.

[8] Tang W, Zhang H, Wang L, Qian H, Qi X (2015). Targeted separation of antibacterial peptide from protein hydrolysate of anchovy cooking wastewater by equilibrium dialysis. Food Chemist.

[9] Athmani N, Dehiba F, Allaoui A, Barkia A, Bougatef A, Lamri-Senhadji MY (2015). Sardina pilchardus and Sardinella aurita protein hydrolysates reduce cholesterolemia and oxidative stress in rat fed high cholesterol diet. J Exp Integr Med.

[10] Chiang W-D, Huang CY, Paul CR, Lee Z-Y, Lin W-T (2016). Lipolysis stimulating peptides of potato protein hydrolysate effectively suppresses high-fat-diet-induced hepatocyte apoptosis and fibrosis in aging rats. Food Nutr Res.

[11] Vik R, Tillander V, Skorve J, Vihervaara T, Ekroos K, Alexson SE (2015). Three differently generated salmon protein hydrolysates reveal opposite effects on hepatic lipid metabolism in mice fed a high-fat diet. Food Chemist.

[12] Huang F, Wang J, Yu F, Tang Y, Ding G, Yang Z (2018). Protective effect of meretrix meretrix oligopeptides on high-fat-diet-induced non-alcoholic fatty liver disease in mice. Mar Drugs.

[13] Abbate JM, Macrì F, Capparucci F, Iaria C, Briguglio G, Cicero L (2020). Administration of protein hydrolysates from anchovy (engraulis encrasicolus) waste for twelve weeks decreases metabolic dysfunction-associated fatty liver disease severity in ApoE–/–Mice. Animals.

[14] FAO ( 2018). Global aquaculture production.

[15] Sachindra N, Bhaskar N, Mahendrakar N (2006). Recovery of carotenoids from shrimp waste in organic solvents. Waste Manag.

[16] Nikoo M, Xu X, Regenstein JM, Noori F (2021). Autolysis of pacific white shrimp (Litopenaeus vannamei) processing by-products: enzymatic activities, lipid and protein oxidation, and anti-oxidant activity of hydrolysates. Food Biosci.

[17] Chen H, Tsai T, Tsai Y, Liao J, Yen C, Chen C (2016). Kefir peptides prevent high-fructose corn syrup-induced non-alcoholic fatty liver disease in a murine model by modulation of inflammation and the JAK2 signaling pathway. Nutr Diabetes.

[18] Nasri R, Abdelhedi O, Jemil I, Daoued I, Hamden K, Kallel C (2015). Ameliorating effects of goby fish protein hydrolysates on high-fat-high-fructose diet-induced hyperglycemia, oxidative stress and deterioration of kidney function in rats. Chem Biol Interact.

[19] Pakfetrat A, Dalirsani Z, Hashemy SI, Ghazi A, Mostaan LV, Anvari K (2018). Evaluation of serum levels of oxidized and reduced glutathione and total anti-oxidant capacity in patients with head and neck squamous cell carcinoma. J Cancer Res Ther.

[20] Zeb A, Ullah F (2016). A simple spectrophotometric method for the determination of thiobarbituric acid reactive substances in fried fast foods. J Anal Methods Chem.

[21] Sun Y, Oberley LW, Li Y (1988). A simple method for clinical assay of superoxide dismutase. Clin Chem.

[22] Classics Lowry O, Rosebrough N, Farr A, Randall R (1951). Protein measurement with the folin phenol reagent. J Biol Chem.

[23] Boonloh K, Kukongviriyapan V, Kongyingyoes B, Kukongviriyapan U, Thawornchinsombut S, Pannangpetch P (2015). Rice bran protein hydrolysates improve insulin resistance and decrease pro-inflammatory cytokine gene expression in rats fed a high carbohydrate-high fat diet. Nutrients.

[24] Matthews DR, Hosker J, Rudenski A, Naylor B, Treacher D, Turner R (1985). Homeostasis model assessment: insulin resistance and β-cell function from fasting plasma glucose and insulin concentrations in man. Diabetologia.

[25] Gharehbagh SA, Azar JT, Razi M (2021). ROS and metabolomics-mediated autophagy in rat’s testicular tissue alter after exercise training; evidence for exercise intensity and outcomes. Life Sci.

[26] Brunt EM, Janney CG, Di Bisceglie AM, Neuschwander-Tetri BA, Bacon BR (1999). Nonalcoholic steatohepatitis: a proposal for grading and staging the histological lesions. Am J Gastroenterol.

[27] Adams L, Angulo P (2006). Treatment of non-alcoholic fatty liver disease. Postgrad Med J.

[28] Ipsen DH, Lykkesfeldt J, Tveden-Nyborg P (2018). Molecular mechanisms of hepatic lipid accumulation in non-alcoholic fatty liver disease. Cell Mol Life Sci.

[29] Rao MS, Reddy JK (2001). Peroxisomal β-oxidation and steatohepatitis. Seminars in liver disease.

[30] Matsuzawa N, Takamura T, Kurita S, Misu H, Ota T, Ando H (2007). Lipid‐induced oxidative stress causes steatohepatitis in mice fed an atherogenic diet. Hepatology.

[31] Chen C, Su X, Hu Z (2019). Immune promotive effect of bioactive peptides may be mediated by regulating the expression of SOCS1/miR155. Exp Ther Med.

[32] Aloysius TA, Carvajal AK, Slizyte R, Skorve J, Berge RK, Bjørndal B (2019). Chicken protein hydrolysates have anti-inflammatory effects on high-fat diet induced obesity in mice. Medicines.

[33] Lemus-Conejo A, Millan-Linares MdC, Toscano R, Millan F, Pedroche J, Muriana FJ (2020). GPETAFLR, a peptide from Lupinus angustifolius L prevents inflammation in microglial cells and confers neuroprotection in brain. Nutr Neurosci.

[34] Lemus-Conejo A, Grao-Cruces E, Toscano R, Varela LM, Claro C, Pedroche J (2020). A lupine (Lupinus angustifolious L peptide prevents non-alcoholic fatty liver disease in high-fat-diet-induced obese mice. Food Funct.

[35] Bujanda L, Hijona E, Larzabal M, Beraza M, Aldazabal P, García-Urkia N (2008). Resveratrol inhibits nonalcoholic fatty liver disease in rats. BMC Gastroenterol.

[36] Martin K, Pritchett J, Llewellyn J, Mullan AF, Athwal VS, Dobie R (2016). PAK proteins and YAP-1 signalling downstream of integrin beta-1 in myofibroblasts promote liver fibrosis. Nat Commun.

[37] Chitapanarux T, Tienboon P, Pojchamarnwiputh S, Leelarungrayub D (2009). Open‐labeled pilot study of cysteine‐rich whey protein isolate supplementation for nonalcoholic steatohepatitis patients. J Gastroenterol Hepatol.

[38] Hamad EM, Taha SH, Abou Dawood A-GI, Sitohy MZ, Abdel-Hamid M (2011). Protective effect of whey proteins against nonalcoholic fatty liver in rats. Lipids Health Dis.

[39] Marnett LJ (1999). Lipid peroxidation—DNA damage by malondialdehyde. Mutat Res.

[40] Sarmadi BH, Ismail A (2010). Anti-oxidative peptides from food proteins: a review. Peptides.

[41] Xiong J-P, Long J-Y, Xu W-Y, Bian J, Huang H-C, Bai Y (2019). Albumin-to-alkaline phosphatase ratio: a novel prognostic index of overall survival in cholangiocarcinoma patients after surgery. World J Gastrointest Oncol.

[42] Khodadoostan M, Shariatifar B, Motamedi N, Abdolahi H (2016). Comparison of liver enzymes level and sonographic findings value with liver biopsy findings in nonalcoholic fatty liver disease patients. Adv Biomed Res.

[43] Caúla A, Lira‐Junior R, Tinoco E, Fischer R (2015). Serum creatinine and alkaline phosphatase levels are associated with severe chronic periodontitis. J Periodontal Res.

[44] Burski K, Ueland T, Maciejewski R (2004). Serum amylase activity disorders in the course of experimental diabetes in rabbits. Veterinarni Medicina.

[45] Huang Y-L, Ma M-F, Chow C-J, Tsai Y-H (2017). Angiotensin I-converting enzyme inhibitory and hypocholesterolemic activities: effects of protein hydrolysates prepared from Achatina fulica snail foot muscle. Int J Food Prop.

[46] Mohamed RS, Marrez DA, Salem SH, Zaghloul AH, Ashoush IS, Farrag ARH (2019). Hypoglycemic, hypolipidemic and anti-oxidant effects of green sprouts juice and functional dairy micronutrients against streptozotocin-induced oxidative stress and diabetes in rats. Heliyon.

[47] Liu M, Wang Y, Liu Y, Ruan R (2016). Bioactive peptides derived from traditional chinese medicine and traditional Chinese food: a review. Food Res Int.

[48] Drotningsvik A, Mjøs SA, Pampanin DM, Slizyte R, Carvajal A, Remman T (2016). Dietary fish protein hydrolysates containing bioactive motifs affect serum and adipose tissue fatty acid compositions, serum lipids, postprandial glucose regulation and growth in obese Zucker fa/fa rats. Br J Nutr.

[49] Drotningsvik A, Mjøs SA, Høgøy I, Remman T, Gudbrandsen OA (2015). A low dietary intake of cod protein is sufficient to increase growth, improve serum and tissue fatty acid compositions, and lower serum postprandial glucose and fasting non-esterified fatty acid concentrations in obese Zucker fa/fa rats. Eur J Nutr.

[50] Sarteshnizi RA, Sahari MA, Gavlighi HA, Regenstein JM, Nikoo M, Udenigwe CC (2021). Influence of fish protein hydrolysate-pistachio green hull extract interactions on anti-oxidant activity and inhibition of α-glucosidase, α-amylase, and DPP-IV enzymes. LWT.

[51] Das J, Ghosh S, Sil PC (2020). Taurine and cardiac oxidative stress in diabetes. Diabetes.

[52] Kulakowski EC, Maturo J (1984). Hypoglycemic properties of taurine: not mediated by enhanced insulin release. Biochem Pharmacol.

[53] Sampath W, Rathnayake R, Yang M, Zhang W, Mai K (2020). Roles of dietary taurine in fish nutrition. Marine Life Sci Technol.

[54] Yang L, Li P, Fu S, Calay ES, Hotamisligil GS (2010). Defective hepatic autophagy in obesity promotes ER stress and causes insulin resistance. Cell Metab.

[55] Madrigal-Matute J, Cuervo AM (2016). Regulation of liver metabolism by autophagy. Gastroenterology.

[56] Czaja MJ (2010). Autophagy in health and disease 2 regulation of lipid metabolism and storage by autophagy: pathophysiological implications. Am J Physiol Cell Physiol.

[57] Ryter SW, Mizumura K, Choi AM (2014). The impact of autophagy on cell death modalities. Int J Cell Biol.

[58] Liu WJ, Ye L, Huang WF, Guo LJ, Xu ZG, Wu HL (2016). P62 links the autophagy pathway and the ubiqutin–proteasome system upon ubiquitinated protein degradation. Cell Mol Biol Lett.

[59] Koga H, Kaushik S, Cuervo AM (2010). Altered lipid content inhibits autophagic vesicular fusion. FASEB J.

[60] Gonzalez-Rodriguez A, Mayoral R, Agra N, Valdecantos M, Pardo V, Miquilena-Colina M (2014). Impaired autophagic flux is associated with increased endoplasmic reticulum stress during the development of NAFLD. Cell Death Dis.

